# Plasma membrane vesicles from cauliflower meristematic tissue and their role in water passage

**DOI:** 10.1186/s12870-020-02778-6

**Published:** 2021-01-07

**Authors:** Paula Garcia-Ibañez, Juan Nicolas-Espinosa, Micaela Carvajal

**Affiliations:** Aquaporins Group, Centro de Edafología y Biología Aplicada del Segura, CEBAS-CSIC, Campus Universitario de Espinardo-25, E-30100 Murcia, Spain

**Keywords:** Plasma membrane, Aquaporin, Brassica, Osmotic permeability

## Abstract

**Background:**

Cauliflower (*Brassica oleracea* L. var. *botrytis*) inflorescences are composed mainly of meristematic tissue, which has a high cellular proliferation. This considerable cellular density makes the inflorescence an organ with a large proportion of membranes. However, little is known about the specific role of the lipid and protein composition of the plasma membrane present in this organ.

**Results:**

In this work, we analyzed the lipids and proteins present in plasma membrane from two different stages of development of cauliflower inflorescence and compared them with leaf plasma membrane. For this purpose, plasma membrane vesicles were obtained by centrifugation for each sample and the vesicular diameter and osmotic permeability (*Pf*) were analyzed by dynamic light scattering and the *stopped-flow* technique, respectively. In addition, fatty acids and sterols were analyzed by gas chromatography and HPLC. The protein composition of the inflorescences and leaves was characterized by HPLC-ESI-QTOF-MS and the data obtained were compared with *Brassicaceae* proteins present in the UniProt database in relation to the presence of aquaporins determined by western blot analysis. The highest *Pf* value was found in 90 day inflorescences-derived plasma membrane vesicles (61.4 ± 4.14 μms^− 1^). For sterols and fatty acids, the concentrations varied according to the organ of origin. The protein profile revealed the presence of aquaporins from the PIP1 and PIP2 subfamilies in both inflorescences and leaves.

**Conclusion:**

This study shows that the composition of the sterols, the degree of unsaturation of the fatty acids, and the proteins present in the membranes analyzed give them high functionality for water passage. This represents an important addition to the limited information available in this field.

**Supplementary Information:**

The online version contains supplementary material available at 10.1186/s12870-020-02778-6.

## Background

Nowadays, crops from the *Brassicaceae* family are among the ones most cultivated worldwide. Of these, the cauliflower (*Brassica oleracea* L. var. *botrytis*) stands out for its high production and economic relevance. For example, about 26 million tons were produced along with broccoli in 2018 (http://faostat.fao.org/). The main reason for its great demand is the presence of diverse health-promoting bioactives - such as glucosinolates, polyphenols, or vitamin C - that add great nutritional value to its edible part [1].

However, little recent information on the phytochemistry of the cauliflower inflorescence molecular structures is available. Smyth [2] described how this inflorescence begins to develop with the formation of secondary meristems. After that, a continuous proliferation of meristematic tissue takes place, generating a highly branched compact pattern with a whitish color and spiral-like shape [2]. Following this, the immature inflorescence initiates its maturation, finally reaching total floral differentiation [3]. Although the intervention of genes related to the identity of the floral meristem, such as *TFL1, LFY, AP1*, or *CAL*, has been studied [4], further comprehension of the biochemical mechanisms underlying this process is needed. In addition, Grevsen et al. [5] observed that environmental factors, mainly temperature, influence the generation of the inflorescences.

As the main focus of the research to date has been the genetic control of the inflorescence development, little is known about the lipid and protein composition of the membranes existing in it. However, a high cellular proliferation is present in the edible part of cauliflower, giving a high membrane concentration [6]. Biological membranes are relevant to cell functionality, not only for their role of compartmentalization, but also by their ability to create an adequate environment for diverse types of proteins, such as transmembrane proteins. The latter mainly determine the specific functionality of each type of membrane present in cells and control diverse constitutive functions, such as endocytosis and ion and water transport [7].

Among the important transmembrane proteins, aquaporins are one of the main protagonists, since they are responsible for the water transport through biological membranes [8]. In cauliflower meristematic tissue cells, it has been observed that there is a high abundance of aquaporins embedded in the vacuolar membrane, which allows swelling of growing cells, while maintaining the cellular turgor [9]. However, the functionality of these proteins can be affected by the lipids of the membrane in which they are embedded [10, 11]. This suggests that the lipid composition of the membrane determines not only its physical characteristics but also the activity and functionality of the proteins present in it [12]. One of the main components of lipid membranes are sterols. These molecules have been linked with different functions, such as lipid packing, since they are able to interact with membrane proteins and fatty acids [13]. Furthermore, membrane sterols have been documented to regulate membrane water permeability [14]. For example, it has been reported that the presence of cholesterol in the bilayer makes it less permeable to water [15]. On the other hand, the composition of fatty acids in cellular membranes also contributes to the thickness, stability, and permeability due to its degree of unsaturation [16].

Therefore, both the lipid and the protein composition are determinants of the membrane transport activity, giving each type of membrane specific functional characteristics. For the above reasons, and with the aim of determining the specific functions of the plasma membrane of the cauliflower meristematic tissue, in this work the presence of fatty acids, sterols, aquaporins, and other proteins was analyzed in two different stages of inflorescence development. To allow a comparison with vegetative tissue, leaf plasma membranes were also analyzed.

## Methods

### Plant material

Fifty commercial cauliflower (*Brassica oleracea* L. var. *botrytis*) seeds from the Whiton cultivar (CAU02417, provided from Sakata Seed Iberica S.L.U., Valencia, Spain) were induced to germinate by imbibition with water and continuous aeration for 24 h. Then, the seeds were transplanted to vermiculite and were kept in darkness, at 28 °C and 60% relative humidity, for 2 days. The seedlings (5 days old) were transferred to the agricultural soil of an experimental farm (37°47′52.7″N, 0°52′00.7″W, 15 m asl, Murcia, Spain) to be cultured in accordance with local legislation. The experiment was carried out from December to February with average temperatures and relative humidities of 17 °C and 60% (day), and 4 °C and 65% (night), under a semi-arid Mediterranean climate. The daily average temperature and relative humidity were recorded with dataloggers (AFORA S.A., Barloworld Scientific, Murcia, Spain). All plants were drip-irrigated with ¼-strength Hoagland nutrient solution. They were harvested at 70 and 90 days after transplanting. The intermediate leaves and inflorescences (15 of each) were sampled at random and weighed fresh in three technical replicates. After that, samples were kept in storage at 4 °C for 1 day until processing. The whole protocol was performed taking into account the Convention on International Trade in Endangered Species of Wild Fauna and Flora (CITES) [17].

### Plasma membrane extraction

Plasma membrane isolation was performed as described in Casado-Vela et al. [18]. Samples of fresh material (100 g) were sliced in small pieces and vacuum-infiltrated with a 1:1.6 (w/v) proportion of an extraction buffer (0.5 M sucrose, 1 mM DTT, 50 mM HEPES, 1.30 mM ascorbic acid, pH 7.5) and 0.5 g of PVP (polyvinylpyrrolidone). After 10 min, samples were homogenized and filtered through a nylon mesh with a pore diameter of 100 μm. Then, the filtrate was centrifuged at 10000×*g* for 30 min, at 4 °C; the supernatants were collected and centrifuged for 35 min at 50000×*g*, at 4 °C. The pellet obtained was resuspended in 500 μl of a buffer containing 5 mM PBS and 0.5 M sucrose (pH 6.5) (FAB). Three different extractions per sample type were performed. Two milliliters of this microsomal fraction were introduced in a two-phase system composed of PEG-3350/Dextran-T500–6.3% (w/w), 5 mM KCl, 330 mM sucrose, 2.5 mM NaF, and 5 mM K_3_PO_4_ (pH 7.8). The system was centrifuged for 5 min at 4000*×g*. Then, the upper phase was collected and washed with a buffer containing 9 mM KCl, 0.2 M EGTA, 0.5 mM NaF, and 10 mM Tris-borate (pH 8.3). Then, a centrifugation at 55000*×g* for 35 min, at 4 °C, was performed. The pellet obtained was resuspended in FAB. The final protein concentration was determined using an RC DC protein assay kit (BioRad, California, USA), with bovine serum albumin as the standard. The purity of the PM was estimated as described in Casado-Vela et al. [18] (Table S1).

### Vesicle size

The mean size of the vesicles obtained from different samples was determined by dynamic light scattering, using a Malvern ZetaSizer Nano XL (Malvern Instruments Ltd., Orsay, France) as described by Barrajón-Catalán et al. [19]. This instrument allows the analysis of particles with diameters from 1 nm to 3 μm.

### Stopped flow light scattering

These measurements were performed in a PiStar (Applied Photophysics, Leatherhead, UK) spectrophotometer at 20 °C, as described in Maurel [8]. The kinetics of each vesicle volume adjustment were monitored by dynamic light scattering at 90° and with a λ_ex_ of 515 nm. Purified plasma membrane vesicles from each sample type were subjected to a 100x dilution in a buffer with 30 mM KCl and 20 mM Tris-Mes (pH 8.3, final osmolarity of 360 mOsmol kg^− 1^ H_2_O). For the measurement, the diluted vesicle preparation was mixed in a 1:1 proportion (v:v) with the same buffer supplemented with 540 mM sucrose (630 mOsmol kg^− 1^ H_2_O). In this way, an osmotic gradient of 270 mOsmol kg^− 1^ H_2_O was generated. The osmotic permeability (*Pf*) was calculated using this formula:

$$ Pf=\frac{K_{exp}\ {V}_0}{A_v\ {V}_w\ {C}_{out}} $$

Where K_exp_ is the adjusted exponential velocity constant, V_0_ is the mean vesicular volume, A_v_ is the mean vesicular surface area, V_w_ is the water molar mass, and C_out_ is the external osmolarity.

### Lipids and sterols analysis

Five hundred microliters of plasma membrane were mixed with a chloroform-methanol (1:2) mixture [20]. As an internal standard for further sterol analysis, β-colestanol (20 μl, at 0.1 mg ml^− 1^) was added. Then, 0.25 ml of chloroform was added to the mixture before centrifugation at 10000*×g* for 6 min. The resultant interphase, corresponding to the protein content, was collected for further proteomic analysis. The chloroformic phase was removed to another tube and evaporated with N_2_. For sterol analysis, 50 μl samples of the chloroformic phase were dried with N_2_ and then acetylated using pyridine (50 μl) and Ac_2_O (100 μl). After 2 h, the solvents were evaporated with N_2_ and 20 μl of ethyl acetate were added. Sterols and fatty acids were determined by gas chromatography, employing an HP5 capillary column (30 m × 0.25 mm × 0.25 μm). This was coupled to a flame ionization detector (FID). Helium was used as the mobile phase (1 ml min^− 1^) and a heat gradient was imposed: from 150 to 195 °C, increasing 3 °C per min, then from 195 to 220 °C at 2 °C per min, and from 220 to 300 °C at 6 °C per min.

### Proteomic analysis

Samples were processed following the method described in Stetson et al. [21]. The isolated plasma membrane proteins from 500 μl of plasma membrane vesicles were mixed with 100 μl of 50 mM ammonium bicarbonate (pH 8.3) with 0.01% Protease Max (Promega, Madison, USA). Then, the samples were reduced by adding 100 μl of 20 mM DTT at 56 °C, for 20 min. After that, alkylation was performed by incubation with 100 μl of 100 mM IAA for 30 min, at room temperature and in the dark. Digestion was performed by incubation with 1 μg of trypsin (1:100 w/w) for 3 h, at 37 °C. The samples were dried in a speed vacuum concentrator. The dry samples were resuspended in 20 μl of water/acetonitrile/formic acid (94.9:5:0.1). Then, they were injected onto an Agilent Advance Bio Peptide Mapping HPLC column (2.7 μm × 100 mm × 2.1 mm, Agilent technologies) thermostatted at 55 °C and with a flow rate of 0.4 ml/min. A mixture of water/acetonitrile/formic acid (10:89.9:0.1) was used as the eluent. For detection, an Agilent 6550 Q-TOF coupled with a dual electrospray (AJS-Dual ESI) was used. The experimental parameters were set in MassHunter Workstation Data Acquisition software (Agilent Technologies, Santa Clara, CA, USA), as described in Martínez-Ballesta et al. [22]. The data were processed with Spectrum Mill MS Proteomics Workbench (Agilent Technologies).

The data obtained were compared with the information available in the UniProt database (www.uniprot.org) for the *Brassicaceae* family. Protein function and location were determined from the Gene Ontology database [23].

### Gel electrophoresis and immunoblotting

Plasma membrane isolated from cauliflower leaves and inflorescences was employed. Ten micrograms of protein per lane were loaded for 12% sodium dodecyl sulfate-polyacrylamide gel electrophoresis (SDS-PAGE), as shown in Muries et al. [24]. Then, the proteins were transferred to a PVDF membrane and maintained for 20 min at 15 V in an electrophoretic transfer cell (Trans-Blot SD cell, BioRad, CA, USA), using Towing transfer buffer supplemented with 0.05% SDS [24]. Blocking solution (TBS containing 2% (w/v) skimmed dry milk) was applied to the membrane for 1 h at room temperature. After that, the membrane was again incubated for 1 h at room temperature, with TBS containing 0.05% Tween 20 and one of the selected antibodies. An antibody raised against the first 45 N-terminus residues of *Arabidopsis thaliana* PIP1;1 (dilution 1:3000, kindly provided by Prof. Dr. Anthony Schäffner) and another raised against 17 residues from the C-terminal peptide of PIP2;2 of *A. thaliana* (dilution 1:20000, kindly provided by Dr. Veronique Santoni) were used. Incubation was performed overnight at 4 °C. Goat anti-rabbit IgG coupled to horseradish peroxidase was employed as a secondary antibody (dilution 1:20000). A chemiluminescent signal was developed with West-Pico Super Signal substrate (Pierce, Rockford, IL, USA). The quantification was carried out using ImageJ software and by performing a densitometry analysis.

### Data analysis

The statistical analysis comprised a one-way ANOVA followed by a Tukey HSD post hoc test, performed using RStudio (version 3.4.4.).

## Results

### Mean size of plasma membrane vesicles

**Fig. 1:**
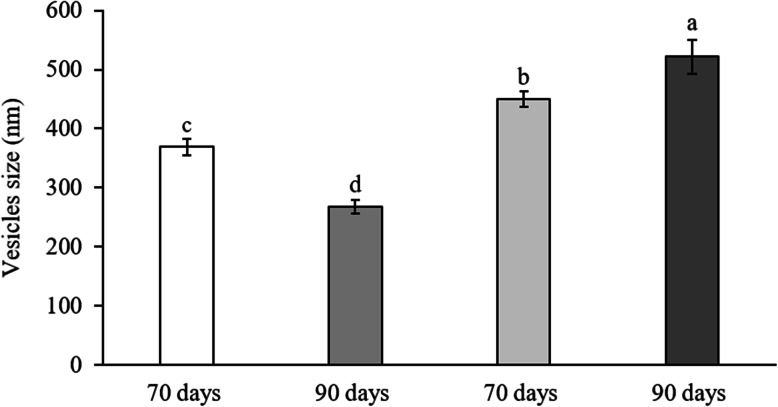
Comparative size (nm) of plasma membrane vesicles obtained from 70- and 90-day cauliflower inflorescences and leaves. The data are represented as the means (*n* = 3, where *n* = different plasma membrane extractions) ± SE. Different letters show statistically significant differences (*p* < 0.05)

**Fig. 2:**
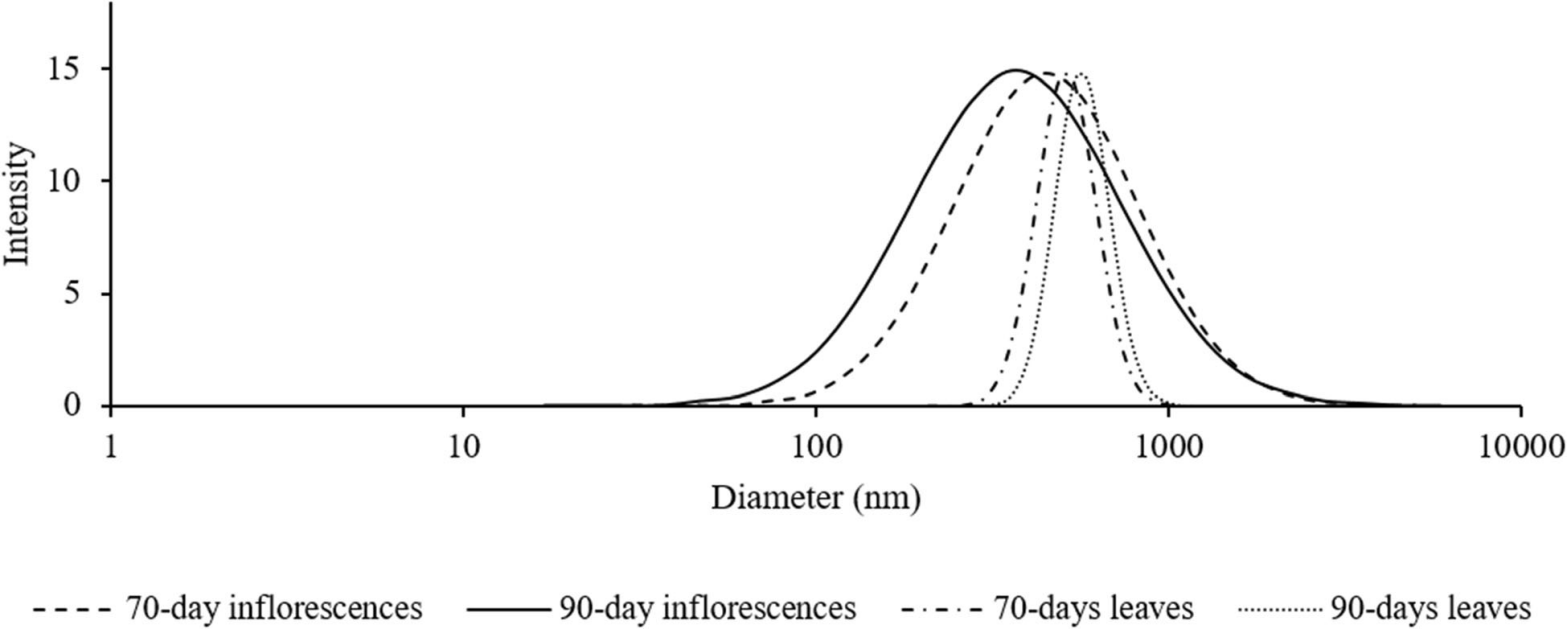
Polydispersity curves of plasma membrane vesicles obtained from 70- and 90-day cauliflower inflorescences and leaves. The curves are mean values (*n* = 3, where *n* = different plasma membrane extractions). X axis is represented in logarithmic scale

The results for the mean vesicle size (nm), represented in Fig. 1, show significant differences between inflorescences and leaves and between the maturation stages (*p <* 0.05). The mean vesicle size was greater for leaves-derived plasma membrane vesicles than for inflorescences-derived ones (*p* < 0.05). The polydispersity data (Fig. 2) show that the variability in the size of inflorescences-derived plasma membrane vesicles was higher than for leaves-derived ones (*p <* 0.05). Hence, vesicles obtained from leaves were more homogeneous in size.

### Osmotic water permeability

**Fig. 3:**
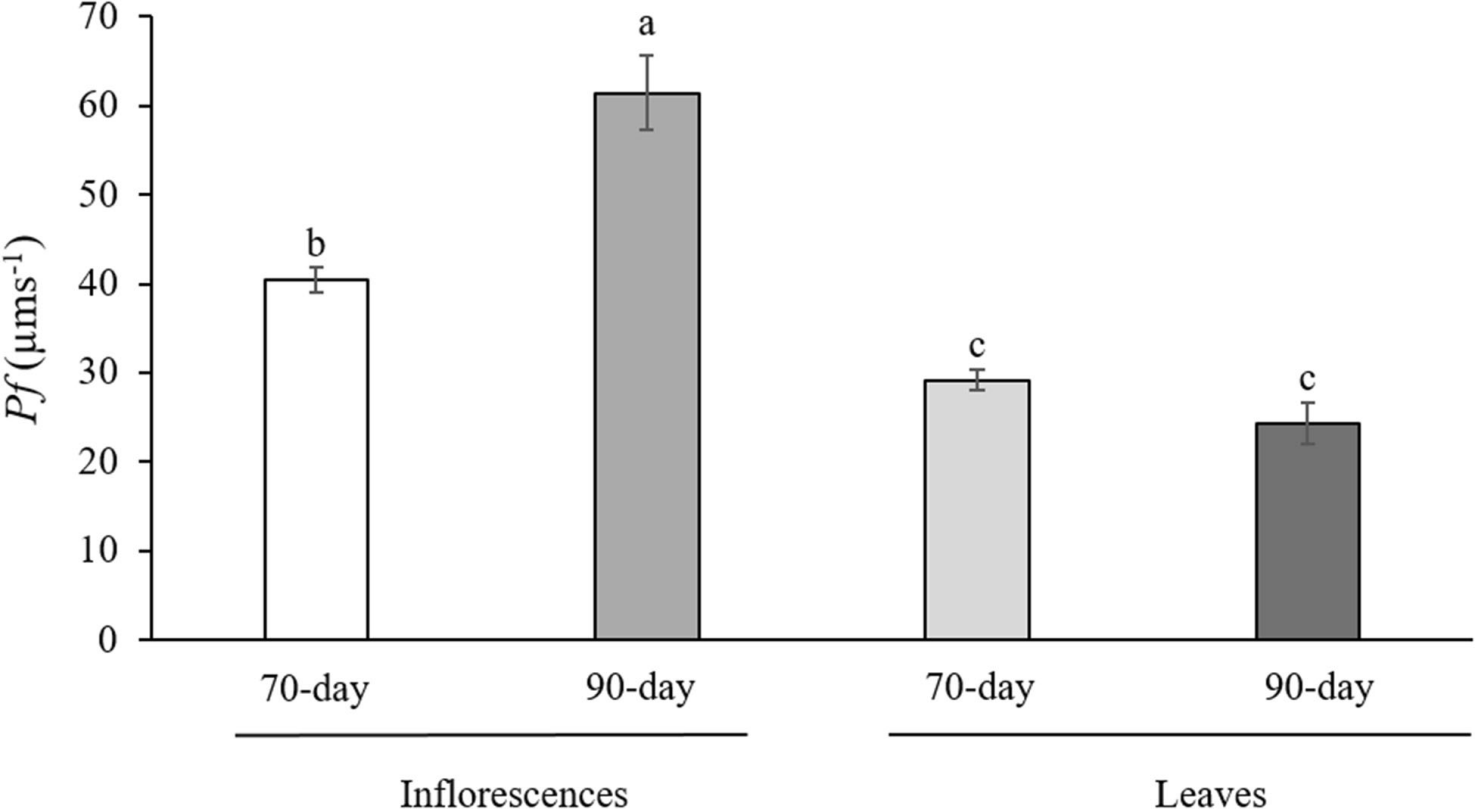
Osmotic water permeability (*Pf*, μm s^− 1^) of plasma membrane vesicles obtained from 70-day and 90-day cauliflower inflorescences and leaves. The data are the means (*n* = 3, where *n* = different plasma membrane extractions) ± SE. Different letters indicate statistically significant differences (*p* < 0.05, Tukey’s test) between treatments

Figure 3 shows that the osmotic water permeability (*Pf,* μm s^− 1^) of the plasma membrane vesicles differed significantly between the two maturation stages of the inflorescences (*p* < 0.05), those derived from 90-day inflorescences having the highest *Pf* (64.4 ± 4.14 μm s^− 1^). Lower values were obtained for leaves, with no significant differences between the two maturation stages (*p* > 0.05).

### Lipid analysis

#### Fatty acids

The results of the fatty acids (% of total fatty acids) analysis are shown in Table 1. The percentage of palmitoleic acid (C16:1) did not vary between 70 days and 90 days for inflorescences-derived plasma membrane vesicles (*p* > 0.05). Furthermore, a similar percentage of palmitoleic acid was found in vesicles derived from 90-day leaves (*p* > 0.05), but a significant decrease was observed for vesicles from 70-day leaves (~ 29–31% vs ~ 19%). For oleic acid (C18:1), similar percentages were found in vesicles derived from inflorescences at the two maturation stages (*p* > 0.05). Surprisingly, the presence of oleic acid in leaves-derived vesicles greatly differed, the percentage in 70-day leaves being double that in inflorescences and almost 14-times higher when compared to 90-day leaves (*p* < 0.05). For linoleic acid (C18:2), a statistically significant decrease was found between day 70 and day 90 for inflorescences-derived plasma membrane vesicles (*p* < 0.05). However, the opposite was observed in leaves-derived vesicles, the content of linoleic acid being increased at 90-days (*p* < 0.05). The linolenic acid (C18:3) percentage differed significantly among the four types of sample (*p* < 0.05). Higher proportions were found in leaves, the vesicles from 70-day leaves having the highest percentage (~ 48%, vs ~ 44% for 90-day leaves). For inflorescences-derived vesicles, the proportion of linolenic acid was higher in 90-day samples than in those taken at 70 days (*p* < 0.05, ~ 40% vs ~ 35%).

**Table 1:** Fatty acid percentage, percentage of monounsaturated fatty acids (MUFA) double bond index (DBI = ∑ (unsaturated fatty acids x number of double bonds)), ratio of unsaturated fatty acids (RUFA = (C18:2 + C18:3)/(C18:1), for plasma membrane from cauliflower inflorescences and leaves at 70 days and 90 days of development. The data are represented as the means (*n* = 3, where n = different plasma membrane extractions) ± SE. Different letters indicate statistically significant differences (*p* < 0.05, in HSD Tukey’s test) between treatments

	Inflorescences		Leaves	
*% Fatty acids*	70-day	90-day	70-day	90-day
Palmitoleic acid (C16:1)	31.41 ± 0.44a	30.99 ± 1.22a	19.09 ± 0.4b	29.42 ± 0.75a
Oleic acid (C18:1)	6.42 ± 0.70b	5.59 ± 0.78b	11.66 ± 0.51a	0.79 ± 0.07c
Linoleic acid (C18:2)	26.71 ± 1.09a	22.66 ± 0.68b	21.24 ± 0.69b	25.35 ± 1.01a
Linolenic acid (C18:3)	35.46 ± 0.91d	40.91 ± 0.8c	48.08 ± 0.64a	44.44 ± 0.51b
MUFA	37.88 ± 0.26a	36.5 ± 0.45a	30.78 ± 0.27b	30.31 ± 0.47b
RUFA	9.94 ± 1.13d	11.8 ± 1.64b	5.95 ± 0.12c	89.1 ± 5.76a
DBI	166.28 ± 1.27c	173.74 ± 0.3b	198.65 ± 3.8a	184.82 ± 3.62b

The total percentages of monounsaturated fatty acids (MUFA) and the ratio of unsaturated fatty acids (RUFA) were also determined (Table 1). The MUFA percentages for inflorescences-derived samples were significantly higher (*p <* 0.05) when compared to leaves. About RUFA, the highest ratio was observed in the 90-day leaves derived plasma membranes (*p* < 0.05). As well, a statistically significant increase (*p* < 0.05) in the double bond index (DBI) was found between the 70-day and 90-day inflorescences cauliflower-derived vesicles. Furthermore, the DBI was significantly higher (*p* < 0.05) in samples from 70-day leaves than in those from 90-day leaves.

#### Sterol content

The sterol content (μg mg^− 1^ of protein) of plasma membrane vesicles was also assessed (Table 2). Campesterol concentration showed a statistically significant increase in vesicles derived from 90-day inflorescences, being 3-fold higher than in those from 70-day ones (*p* < 0.05). No differences in the campesterol concentration were found between vesicles derived from 70- and 90-day leaves (*p* > 0.05). For stigmasterol, no statistically significant differences were found between vesicles from inflorescences at the two maturation stages (*p* > 0.05). However, in leaves-derived vesicles, the concentration of stigmasterol was higher for 90-day leaves (0.16 ± 0.07 vs 0.25 ± 0.09 μg mg^− 1^ of protein, *p* < 0.05). A 2-fold, statistically significant increase in β-sitosterol was found in vesicles from 90-day inflorescences when compared to those of 70-day inflorescences (*p* < 0.05). A similar difference was seen when comparing vesicles from 70-day leaves with those of 90-day leaves (0.95 ± 0.11 vs 2.14 ± 0.37 μg mg^− 1^ of protein, *p* < 0.05). The stigmasterol/β-sitosterol ratio was also analysed. The highest ratio was found in vesicles derived from 70-day inflorescences, 2-times higher than for 90-day inflorescences and 5-times higher than for both 70-day and 90-day leaves (*p* < 0.05). A statistically significant difference in the stigmasterol/β-sitosterol ratio was not found between 70-day and 90-day leaves (0.17 ± 0.02 vs 0.12 ± 0.03 μg/mg of protein, *p* > 0.05).

**Table 2:** Sterol content (μg mg^− 1^ of protein) of plasma membrane from cauliflower inflorescences and leaves at 70 days and 90 days of plant development. The data are represented as the means (*n* = 3, where *n* = different plasma membrane extractions) ± SE. Different letters indicate statistically significant differences (*p* < 0.05, HSD Tukey’s test) between treatments

	Inflorescences		Leaves	
*μg mg*^*−1*^ *of protein*	70-day	90-day	70-day	90-day
Campesterol	1.94 ± 0.39b	5.68 ± 0.64a	1.72 ± 0.46b	2.10 ± 0.35b
Stigmasterol	0.53 ± 0.16a	0.60 ± 0.19a	0.16 ± 0.07c	0.25 ± 0.09b
β-Sitosterol	0.79 ± 0.11b	1.71 ± 0.05a	0.95 ± 0.11b	2.14 ± 0.37a
Stigmasterol/β-Sitosterol ratio	0.67 ± 0.08a	0.35 ± 0.1b	0.17 ± 0.02c	0.12 ± 0.03c

#### Proteomic analysis and immunoblotting

A proteomic analysis was performed with the samples of isolated plasma membrane vesicles form inflorescences and leaves in order to assess qualitatively the proteins present in the vesicles obtained for further analysis. As shown in Fig. S1, the proteins obtained were organized according to their cellular characteristics among soluble, membrane and unclassified. In all samples studied (inflorescences and leaves at both maturation stages) the number of soluble proteins identified was higher (40–48%) than the number of membrane proteins (40–44%). Also, high percentages of unclassified proteins were identified in leaves (13–14% in 90-d and 70-d, respectively) than in inflorescence (8.6–8.9% in 90-d and 70-d, respectively). The number of membrane proteins determined in inflorescence was very similar (42%) for both times (90-d and 70-d) while the number of these proteins determined in leaves was higher at 70-d (44%) than at 90-d (40%).

**Fig. 4:**
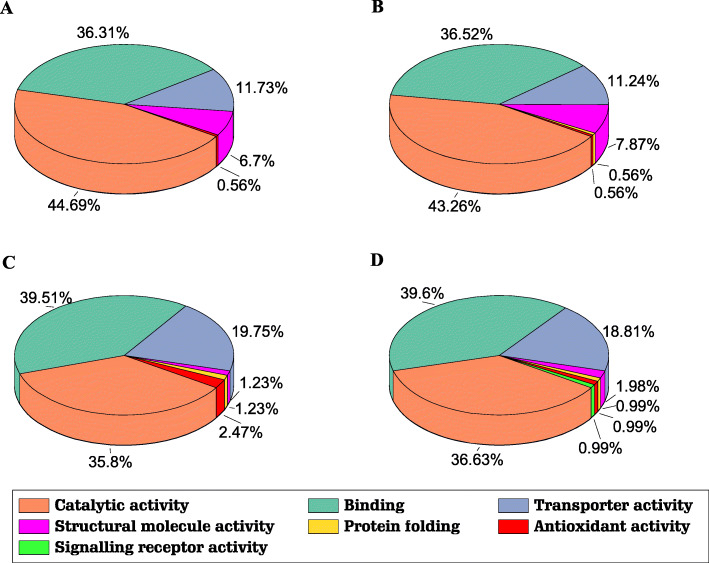
Functional classification of plasma membrane proteins identified in cauliflower. The proteins identified were classified based on seven categories described in the Gene Ontology database [23]. **a** 70-day inflorescences, **b** 90-day inflorescences, **c** 70-day leaves, and **d** 90-day leaves. The figure design has been done with Origin (Pro), Version 2019. OriginLab Corporation, Northampton, MA, USA

Within the plasma membrane proteins, they were also classified by their molecular function (Fig. 4). They were grouped in seven functional clusters (catalytic, structural molecules activity, signalling receptor activity, binding, protein folding, transporter activity and antioxidant activity). All the samples showed the similar protein distribution, being the catalytic (35–44%) and the binding proteins (36–39%) standing out as the main groups, followed by transporter (11–18%). The number of transporter proteins were lower identified in inflorescences (11%) than in leaves (18–19%). The structural proteins were higher in inflorescence (6–7%) than in leaves (0.5%). The rest of the proteins identified were very low in all the samples, appearing the signalling receptor proteins only in 90-d leaves.

A search focused on the aquaporins present in plasma membrane derived from cauliflower inflorescences and leaves was also performed. Aquaporin-related peptides were spotted in all sample types (Table 3). In both inflorescences and leaves, peptides corresponding to a wide group of PIP1 aquaporin subfamilies (PIP1;1, PIP1;2, PIP1;3, PIP1;4, and PIP1;5) were identified. Nevertheless, only in the inflorescence samples were peptides related to the PIP2 subfamily detected, PIP2;5 and PIP2;7. Special emphasis can be placed on PIP2;7, for which three different peptide fragments were detected, while only one peptide determined PIP2;5. Members of the Tonoplast Intrinsic Protein (TIP) subfamily were also identified in both types of sample, although this group of aquaporins is generally targeted to the vacuolar membrane. In particular, TIP1;2 and TIP2;1 were found in 70-day inflorescences and 90-day leaves, while only TIP1;2 peptides were detected in 90-day inflorescences and 70-day leaves samples (Table 3).

**Table 3:** Aquaporin proteins identified in plasma membrane samples of cauliflower inflorescences and leaves. All protein sequences were retrieved from *Brassica oleracea* L. var. *oleracea* information in the NCBI and UniProt databases (ID). The symbols **‘+’** and **‘-’** indicate the presence or absence of the protein in the samples, respectively

Protein	NCBI ID	UniProt ID	Score	Inflorescences		Leaves	
				70-day	90-day	70-day	90-day
PIP1;1	XP_013604594.1	A0A0D3DUU2	31.8	**+**	**+**	**+**	**+**
PIP1;2	XP_013637020.1	A0A0D3C6I1	34.9	**+**	**+**	**+**	**+**
PIP1;3	XP_013600561.1	A0A0D3C6T1	22.3	**+**	**+**	**+**	**+**
PIP1;4	XP_013612790.1	A0A0D3D7M3	22.3	**+**	**+**	**+**	**+**
PIP1;5	XP_013599049.1	A0A0D3DFM5	22.3	**+**	**+**	**+**	**+**
PIP2;5	XP_013599897.1	A0A0D3DT38	11.9	**+**	**+**	–	–
PIP2;7	XP_013629883.1	A0A0D3BL75	27.4	**+**	**+**	–	–
TIP1;2	XP_013613430.1	A0A0D2ZPE6	18.3	**+**	**+**	**+**	**+**
TIP2;1	XP_013587105.1	A0A0D3CJP0	14.7	**+**	–	–	**+**

**Fig. 5:**
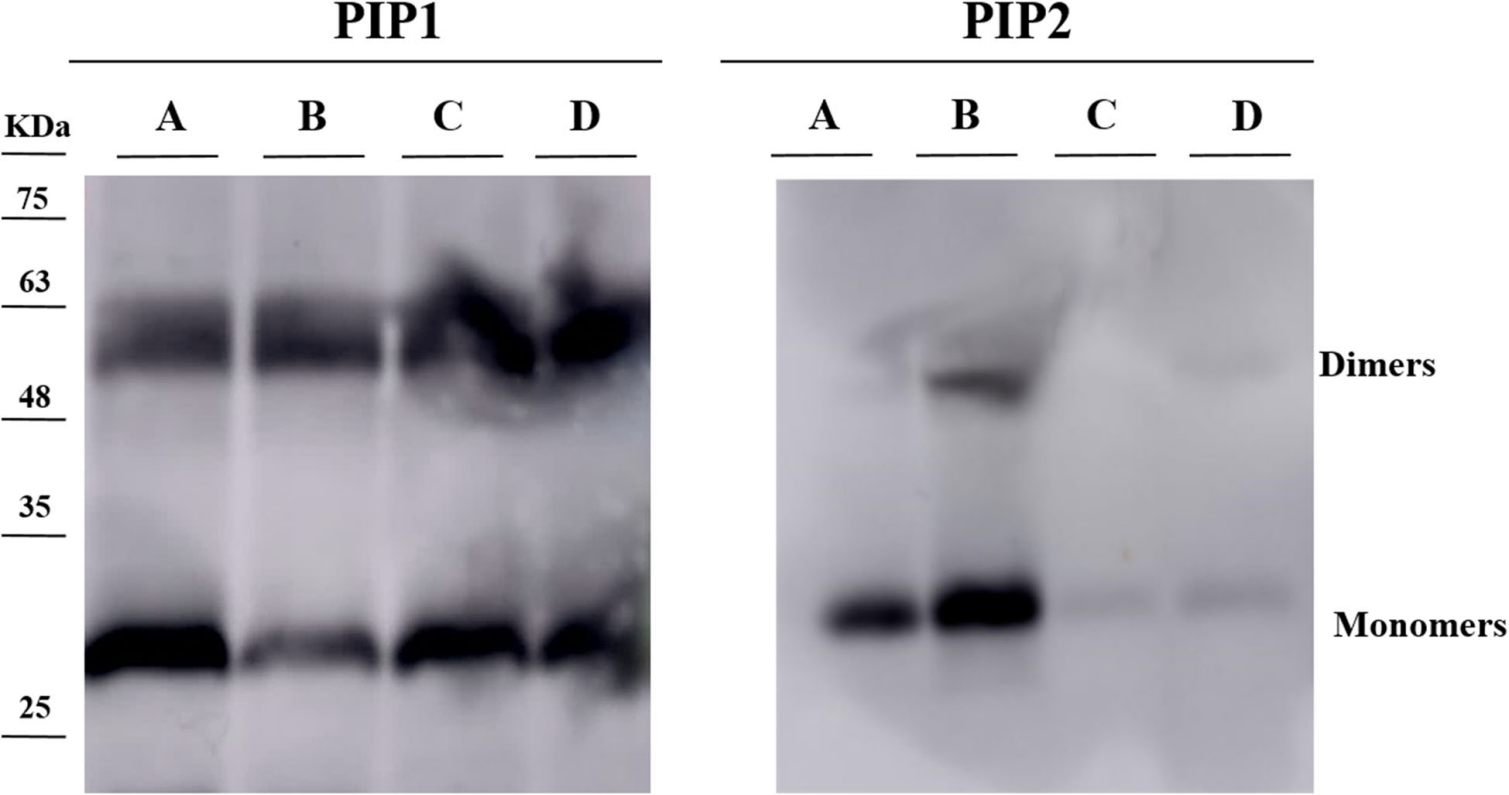
Immunoblotting analysis for PIP1 and PIP2 aquaporins present in cauliflower plasma membrane vesicles. **a** plasma membrane from 70-day inflorescences, **b** plasma membrane from 90-day inflorescences, **c** plasma membrane from 70-day leaves, **d** plasma membrane from 90-day leaves

The results obtained from SDS-PAGE analysis of plasma membrane proteins from leaves and inflorescences are shown in Fig. 5. The presence of two bands was detected; an upper band of 60 kDa, corresponding to dimeric (D) forms of PIPs, and a lower band of ca. 30 kDa, corresponding to the monomeric form (M). Two PIPs groups were analysed, PIP1 and PIP2. For PIP1, bands from samples of 70-day and 90-day inflorescences (23.4 and 15.7% D + M) were less dense than those of 70-day and 90-day leaves-derived plasma membrane proteins (31 and 28.9% D + M). For PIP2 aquaporins, much denser bands were found for 70-day (27.7% D + M) and 90-day inflorescences (61.4% D + M) when compared with 70-day and 90-day leaves-derived samples (5.2 and 5.7% D + M).

## Discussion

The isolation of plasma membrane vesicles using the two-phase aqueous polymer technique [25] has been reported to produce homogeneous material in terms of yield and composition [26]. However, the vesicles isolated from plant tissues can vary depending on the type of plant [20], the organ, and the culture conditions [22]. In our work, the vesicles obtained from adult plants were bigger than those obtained previously from seedlings [22]. Furthermore, the vesicles obtained from inflorescences were smaller but more heterogeneous in size than those obtained from leaves. Also, the vesicles yield from inflorescences was double that from leaves (data not shown). Although these results, that could have been due to differences in cell size and tissue lignification, may be unimportant in a plant physiological study they could be important if an industrial application is considered [27].

One of the main functions of the plasma membrane is the regulation of the passage of diverse molecules and water through it. The *Pf* usually is the parameter chosen to describe water fluxes across the plant membranes isolated vesicles that are driven by the osmolarity gradient [28]. In fact, the osmotic shock applied to our vesicles should not produce any small pore that suppressed convection that have been reported to leaded to a vesicles rupture [29]. The plasma membrane vesicles derived from broccoli leaves by Martínez-Ballesta et al. [30] had *Pf* values similar to the ones obtained in our work. Nevertheless, the *Pf* values obtained for the plasma membrane vesicles derived from 70-day and 90-day inflorescences were 1.6- and 2.5-times higher, respectively, than those of vesicles from 90-day leaves. Since little or no information concerning *Pf* in protoplasts or vesicles derived from *Brassica* inflorescences exists, these results shed light on this matter. Similar values of *Pf* have been reported for plasma membrane vesicles and protoplasts obtained from pepper roots (30 and 40 μm s^− 1^) [31]. Furthermore, *Pf* values as high as 540 μm s^− 1^ have been found in plasma membrane from *Beta vulgaris* roots [32]. This suggests that the water osmotic permeability in inflorescences might be similar to that in roots, due to their requirement for water to maintain turgor. Indeed, a relationship between cell turgor in meristematic tissue and cellular division has been reported [33].

One of the main structural components affecting the physical characteristics of biological membranes are fatty acids. The proportions of different saturated and unsaturated fatty acids may affect the permeability of the bilayer [34]. In our study, the plasma membrane vesicles obtained from cauliflower had a high proportion of unsaturated fatty acids, which provides greater fluidity [35]. In leaf plasma membranes from other species - such as broccoli, *Cakile maritima* L., and *Brassica napus* L. - linolenic acid (C18:3) was a minor component [20]. However, the proportion of this fatty acid was greater in plasma membrane vesicles produced from cauliflower leaves and inflorescences. This difference in fatty acids distribution might have a protective effect against temperature changes, since previous work was carried out in a crop chamber but we grew cauliflowers in the field; an increase in linolenic acid (C18:3) was found in peach fruits under low-temperature stress [36]. Also, a greater degree of unsaturation produces looser packing of the polyunsaturated carbon chains, decreasing the interaction with other molecules and allowing deeper penetration of water into the bilayer [37]; this could be related to the higher *Pf*. But, the fact that *Pf* was higher in 90-day inflorescences than in 70-days inflorescences, must be related to the aquaporins presence. In our vesicles derived from cauliflower leaves the oleic acid (C18:1) proportion was lower than that reported for *Brassica oleracea* L. var. italica in Chalbi et al. [20]. When comparing the data of *B. oleracea* var. italica leaves [20] with our work, a higher RUFA (ratio of unsaturated fatty acids) was obtained in cauliflower, due to the low percentage of oleic acid (5.95 ± 0.13 and 89.1 ± 5.76 for 70 and 90-day leaves vs 1.19 ± 0.18 in broccoli leaves). In the same way, the DBI was also affected by the different proportions of unsaturated fatty acids. In our analysis, the highest DBI was found in 70-day leaves, since the percentage of linolenic acid (C18:3) was higher in this sample than in *B. oleracea* var. italica (48.08 ± 0.64% vs 5.52 ± 0.8%). In previous studies of soy (*Glycine max* L.) plasma membrane, a rise in oleic acid (C18:1) and a decrease in linoleic acid (C18:2) and linolenic acid (C18:3) were observed [38], which could possibly lead to an increase in membrane rigidity. Since the plants studied in Chalbi et al. [20] were grown in a controlled environment chamber, the lipid proportions might be quite different from those of plants cultivated in the field, where the climatic conditions, such as temperature and humidity, are highly variable.

Sterols also contribute to the bilayer permeability [26]. Campesterol has been studied regarding its contribution to increasing the spatial organization of the lipid bilayer and, thus, its order [39]. Furthermore, it has been linked with a decrease in ionic permeability through lipid membranes [40]. In our work, plasma membrane from 90-day cauliflower inflorescences had the highest content of campesterol per mg of protein. This might be due to the dual function of campesterol, as a structural component and also the precursor of brassinosteroid hormones that are required for normal plant development [41]. As has been reported in *Arabidopsis thaliana,* brassinosteroids help root meristem growth [42]. The fact that the stigmasterol/β-sitosterol ratio was much higher in the plasma membranes of inflorescences, mainly in the young ones (70 d), could have contributed to the increase in *Pf*. In fact, sitosterol has been pointed out as the main regulator of water permeability though membranes along with aquaporins [26, 43]. However, the fact that the highest P_f_ value was obtained for 90-day inflorescences, which present a lower sitosterol/stigmasterol ratio provide to aquaporins higher contribution to water transport.

By means of the proteomic analysis performed by HPLC-ESI-QTOF-MS, the whole batch of identified proteins in each sample were analysed and categorized according to their cellular location (Fig. S1). However, a few proteins could not be assigned to defined categories in each fraction due to the lack of information. Although a high percentage of membrane proteins were identified in all samples (42–44%), a notable presence of soluble proteins was also found (40–49%). This could be considered a contamination of the membrane samples, although the number of identified proteins is high, the total amount should be very low.

In addition, we classified proteins based on their functional category within the plasma membrane identified proteins (Fig. 4) [23]. The presence of different transporter activity proteins (11–19%) was detected; these comprised transmembrane ion transporters and ion channels, also located mainly in the plasma membrane. The number of detected transporter proteins revealed a decrease in inflorescence. This could be related with the specialization of the tissue since the meristematic tissue (inflorescence) probably need lower category of plasma membrane transporters.

Table 3 shows the aquaporins identified in plasma membrane extracts of cauliflower inflorescences and leaves. As PIPs aquaporins showed a very conservative structure, the digestion by trypsine enzyme and solubilisation is very similar in all of them. Although the knowledge of the sequences of all *B. oleracea* aquaporins was very useful to investigate the presence of MIPs isoforms, and the analysis of peptides revealed five PIP1 isoforms (PIP1;1, PIP1;2, PIP1;3, PIP1;4, and PIP1;5) (Table 3), due to the high homology within the PIP1 subfamily, we could not identify isoforms unambiguously. Also, two TIPs were identified in our plasma membrane samples. Since TIPs are usually located in the tonoplast [44], their presence could be related with vacuolar contamination. However, the fact that TIPs have been found located in the plasma membrane in pea cotyledons [45] points to the possibility that TIPs were present in our plasma membrane.

Additionally, PIP2 subfamily proteins were found only in inflorescences (Table 3), in particular PIP2;5 and PIP2;7. Of these, only PIP2;7 was unambiguously identified; this aquaporin has been shown to be expressed mainly in *B. oleracea* flowers [46]. Furthermore, when PIP2;7 was overexpressed in *A. thaliana* roots the hydraulic conductivity increased six-fold, showing the important role of PIP2;7 in water transport.

Further information about plasma membrane aquaporins in cauliflower was obtained from immunoblotting (Fig. 5). The results show that PIP1 was present in both leaves and inflorescences, although its density in inflorescences was lower. The greater presence of PIP1 proteins in leaves could be explained by its regulatory role in CO_2_ transport [47]. This CO_2_ transport would be indispensable for the photosynthetic activity in leaves, whereas in inflorescences the constant cell division and growth would produce CO_2_ that would need to be carried to the leaves. In addition, the other PIP1 members have been postulated as O_2_ transport facilitators; in particular, PIP1;3 in tobacco plants [48].

In addition, the results for PIP2 show a greater density in samples from inflorescences - which mirrors the information obtained in the proteomic assay- in which PIP2 could only be detected in those samples (Fig. 5). The exclusive identification of PIP2 proteins in inflorescences (PIP2;1, PIP2;2, PIP2;5, and PIP2;7) could be related to the elevated demand for water in cauliflower inflorescences, to maintain nutrient uptake and turgor [49]. The localization of PIP2 in plasma membrane samples from inflorescences (Fig. 5) could also explain the differences observed in *Pf*; the 90-day inflorescences showed the highest *Pf*, in accordance with the higher concentration of PIP2 found in these samples (Fig. 3). The non-detection of PIP2 in leaves could indicate that the function of PIPs is performed here by TIPs or that only limited water transport across the lipid bilayer occurs. However, this aspect needs to be investigated further.

## Conclusions

In summary, this study shows how the different elements present in the cauliflower inflorescences plasma membrane play a key role in water passage. This membrane is characterized by a low degree of unsaturation, which could increase the rigidity, decreasing water transport, but the high content in sitosterol (highly correlated with water passage) would compensate this fact. In relation to this, the high presence of aquaporins in inflorescences, especially PIP2;5 and PIP2;7, indicates a potential role of aquaporins in the water transport required for the continuous development of the meristematic tissue. Furthermore, the fact that the aquaporins contribution to water transport in inflorescences must be higher than in leaves, with a potential correlation with the stage of development, provides to sterols and aquaporins a specific role in development. Our work highlights the need for further research on specific aquaporins in relation to adult plant development under natural conditions.

## Supplementary Information

**Additional file 1: Figure S1.** Classification of proteins identified in cauliflower plasma membrane isolated vesicles. Proteins identified were classified based on three categories corresponding the information available in the Gene Ontology database [23]. (A) 70-day inflorescences, (B) 90-day inflorescences, (C) 70-day leaves, and (D) 90-day leaves. The figure design has been done with Origin(Pro), Version 2019. OriginLab Corporation, Northampton, MA, USA. **Figure S2.** Raw images of immunoblotting analysis for PIP1 and PIP2 aquaporins present in cauliflower plasma membrane vesicles. A: plasma membrane from 70-day inflorescences, B: plasma membrane from 90-day inflorescences, C: plasma membrane from 70-day leaves, D: plasma membrane from 90-day leaves. **Table S1.** Average of the enzymatic activities (nmol min-1 mg-1 Protein) of plasma membrane and microsomal fractions measured in the purification fraction after aqueous polymer two-phase partitioning method.

## Data Availability

The datasets used and/or analysed during the current study are available from the corresponding author on reasonable request.

## Abbreviations

DBI: Double bond index
ER: Endoplasmic reticulum
FID: Flame ion detector
HPLC: High Performance Liquid Chromatography
MUFA: Monounsaturated fatty acids
PIP: Plasma membrane intrinsic proteins
PVP: Polyvinylpyrrolidone
RUFA: Ratio of unsaturated fatty acids
SDS-PAGE: Sodium dodecyl sulfate-polyacrylamide gel electrophoresis
TBS: Tween 20 blocking solution
TIP: Tonoplast intrinsic protein
