# Supplementary material for: Plasma membrane vesicles from cauliflower meristematic tissue and their role in water passage
Source: BMC Plant Biol. 2021 Jan 7;21:30. doi: 10.1186/s12870-020-02778-6 (PMC7791869; doi:10.1186/s12870-020-02778-6)
Supplement: Supplementary file 1 — Additional file 1: Figure S1. Classification of proteins identified in cauliflower plasma membrane isolated vesicles. Proteins identified were classified based on three categories corresponding the information available in the Gene Ontology database [23]. (A) 70-day inflorescences, (B) 90-day inflorescences, (C) 70-day leaves, and (D) 90-day leaves. The figure design has been done with Origin(Pro), Version 2019. OriginLab Corporation, Northampton, MA, USA. Figure S2. Raw images of immunoblotting analysis for PIP1 and PIP2 aquaporins present in cauliflower plasma membrane vesicles. A: plasma membrane from 70-day inflorescences, B: plasma membrane from 90-day inflorescences, C: plasma membrane from 70-day leaves, D: plasma membrane from 90-day leaves. Table S1. Average of the enzymatic activities (nmol min-1 mg-1 Protein) of plasma membrane and microsomal fractions measured in the purification fraction after aqueous polymer two-phase partitioning method. [file 12870_2020_2778_MOESM1_ESM.docx]

**Fig. S1.** Classification of proteins identified in cauliflower plasma membrane isolated vesicles. Proteins identified were classified based on three categories corresponding the information available in the Gene Ontology database [23]. (A) 70-day inflorescences, (B) 90-day inflorescences, (C) 70-day leaves, and (D) 90-day leaves. The figure design has been done with Origin(Pro), Version 2019. OriginLab Corporation, Northampton, MA, USA.

**Fig.S2.** Raw images of immunoblotting analysis for PIP1 and PIP2 aquaporins present in cauliflower plasma membrane vesicles. A: plasma membrane from 70-day inflorescences, B: plasma membrane from 90-day inflorescences, C: plasma membrane from 70-day leaves, D: plasma membrane from 90-day leaves.

Figure Supplementary 1


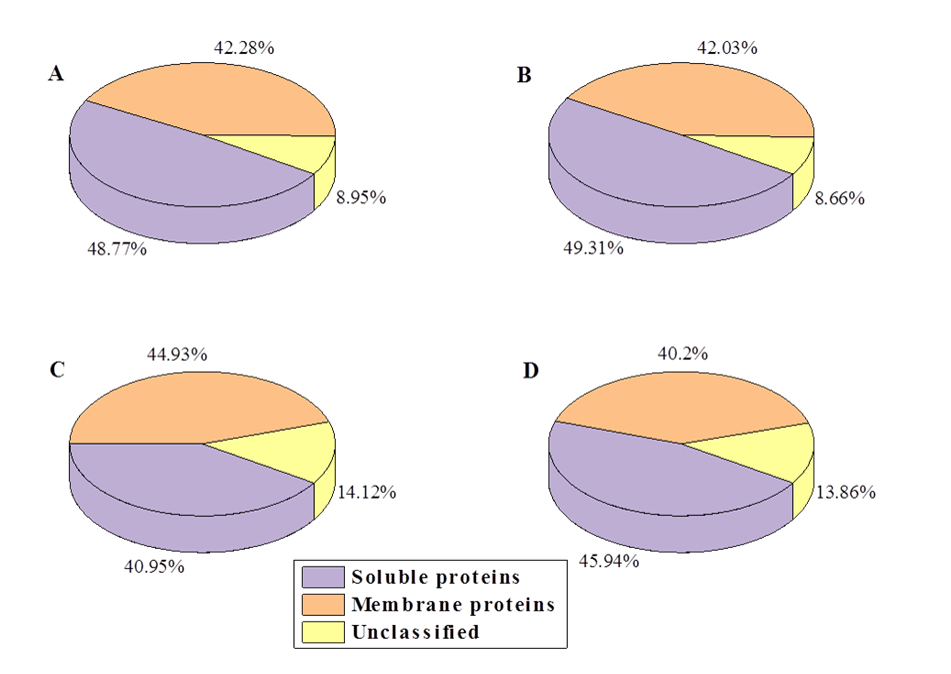


Figure Supplementary 2


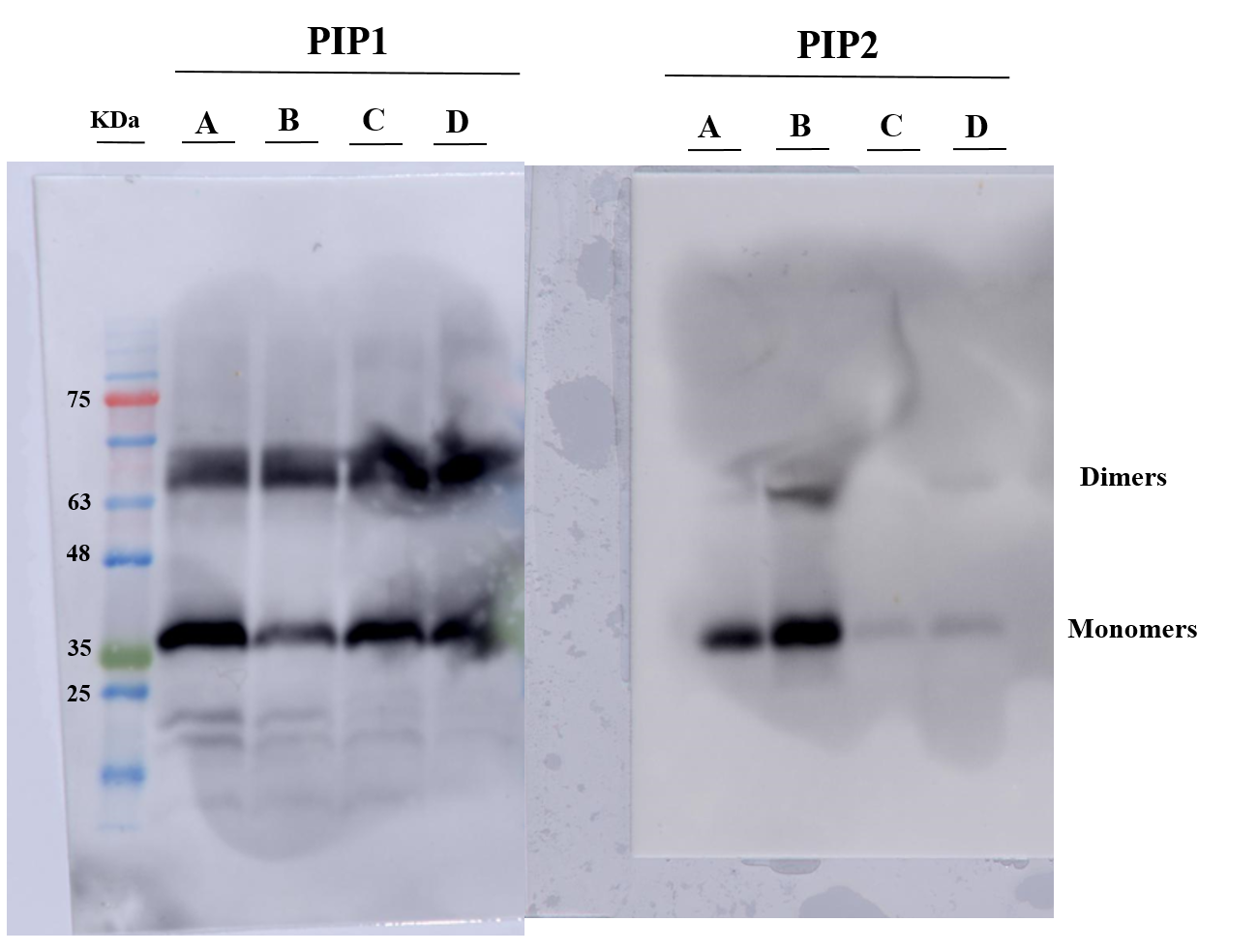


**Table S1.** Average of the enzymatic activities (nmol min-1 mg-1 Protein) of plasma membrane and microsomal fractions measured in the purification fraction after aqueous polymer two-phase partitioning method.

| Enzyme | Organelle | nmol min^-1^ mg^-1^ protein | | Enrichment (%)in the plasma membrane fraction |
| --- | --- | --- | --- | --- |
|  |  | Plasma membrane fraction | Microsomal fraction |  |
| Vanadate-sensitive ATPase | Plasma membrane | 178 ± 20.85 | 13 ± 1.2 | 92.69 |
| Nitrate-sensitive ATPase | Tonoplast | 8.4 ± 3.7 | 255 ± 18.6 | 3.29 |
| Cytochrome C oxidase | Mitochondria | 9.7 ± 6.35 | 1082.5 ± 58.7 | 0.89 |
| Inosine diphosphatase | Golgi apparatus | 3.6 ± 0.6 | 82.6 ± 6.35 | 4.35 |
